# A Meta-Analysis of Transcranial Direct Current Stimulation on Substance and Food Craving: What Effect Do Modulators Have?

**DOI:** 10.3389/fpsyt.2020.00598

**Published:** 2020-06-26

**Authors:** Jiasi Chen, Jingmin Qin, Qinghua He, Zhiling Zou

**Affiliations:** Faculty of Psychology, Southwest University, Chongqing, China

**Keywords:** transcranial direct current stimulation, tDCS, craving, substance dependence, food addiction, dorsolateral prefrontal cortex

## Abstract

Substance addiction and food addiction are significant social problems worldwide. In previous studies of substance addiction, transcranial direct current stimulation (tDCS) has been used to influence craving of substance or food. However, the reported effects are not always consistent due to inconsistent experimental settings. The way modulators influence the effect of tDCS on substance addiction is worth exploring. This meta-analysis was conducted to estimate the effect size of tDCS on substance and food craving and to investigate the influence of potential modulators. We systemically identiﬁed and reviewed studies on substance/food craving using tDCS that were published between January 2008 to January 2020. A total of 32 eligible studies were identified. Hedges' g was computed as an indicator of the effect of tDCS and some potential moderators (substance type, stimulation sites, current intensities, number of sessions, duration of stimulation, and study design) were examined using subgroup analysis. Random effects analysis revealed a total medium effect size [Hedges' *g* = 0.536, 95% confidence interval (CI): 0.389–0.683, after adjusting Hedges' *g* = 0.416, 95% CI: 0.262–0.570] preferring active over sham stimulation to reduce craving. A significant difference was observed between the number of sessions (repeated stimulation was better than single stimulation). The duration of stimulation may have a positive influence on the effects of tDCS. No other significant differences were found in other subgroups analysis. In conclusion, our results provided evidence that tDCS can be an effective way to reduce craving of substance or food, and longer multiple stimulus durations in all can more effectively reduce craving; however, the influences of modulators still need be to be examined in depth in future.

## Introduction

Substance dependence (or substance addiction) is a chronic relapsing brain disorder leading patients to use a substance continuously despite the negative consequences that result from doing it (1). Many types of substances can cause addiction, such as addictive drugs including alcohol and tobacco. According to statistics released by the United Nations Office of Drug and Crimes, the percentage of individuals using cannabis, cocaine, and opioid worldwide were 3.8, 0.37, and 1.08%, respectively, in 2017 (2). Meanwhile, the harmful use of alcohol was estimated to cause 2.5 million deaths each year (3) and tobacco use causes more than five million deaths worldwide each year (4).

In the meantime, food addiction can be described as the dependence on refined foods that meets the Diagnostic and Statistical Manual of Mental Disorders (DSM-IV) substance use disorder criteria (5). Food addiction is significantly linked to obesity (6). Considering around 93.3 million US adults (7) and about 86 million people in China (8) could be classified as obese in 2016, food addiction is similar to substance addiction because food regulates body process by acting as the source of required energy and has a hedonic component that makes it an effective natural reward. Food-related rewards may promote increased intake and trigger withdrawal-related symptoms (e.g., overeating), suggesting that the behaviors parallel substance abuse (9). One possible explanation for food addiction (or overeating) is that sugar, fats, salt, caffeine, refined sweeteners, and refined carbohydrates in processed foods are addictive substances (5).

With substance addiction or food addiction, craving is dependent upon the past experience of an urge or desire to use substances (10). Reducing craving is an important aspect of treating substance dependence or food addiction. A direct method to reduce craving is pharmacotherapy (11–14), which is a long-term therapy focused on substitution or withdrawal. Because the use of medication carries a risk of damaging cardiopulmonary function (15), its application requires care. Cognitive treatments such as cognitive-behavior coping skills treatment (CBT) (16) and psychological counseling (17) are other mainstream treatments for helping patients recognize, avoid, and cope with the substance. It is often used with medications that interact with the type of psychotherapy provided (18, 19). Additionally, with the development of neural science, non-invasive neurostimulation techniques, including transcranial direct current stimulation (tDCS) and transcranial magnetic stimulation (TMS), have been widely used to reduce substance cravings (20, 21). Electromagnetic brain stimulation could regulate activity in specific brain regions. Recent studies have explored the application of non-invasive neurostimulation for the treatment of substance dependence (22–25).

Among them, tDCS, which uses a weak safe current of 1–2 mA for 3–20 min to increase (anodal tDCS) or decrease (cathodal tDCS) cortical excitability (26, 27), has a significant effect on reducing cravings (28, 29). However, the results of the effect of tDCS on drug craving are mixed. Salling et al. (30) reviewed research on brain stimulation in addiction and found that tDCS has an acute effect on drug and alcohol cravings without consistent results. Coles et al. (31) found that tDCS reduced craving and consumption for alcohol and drugs while the results for tobacco were unclear because of different stimulation methods and parameters. Some studies indicated that a single stimulation of the left dorsolateral prefrontal cortex (DLPFC) with anodal tDCS significantly reduced cravings (32–35), but other findings did not support this conclusion (36–38). This kind of inconsistency may come from different study designs, stimulation parameters, and characteristics of participants in different studies.

Considering the inconsistent findings of empirical investigations and that only a medium effect of tDCS was found in a meta-analysis, it is essential to explore potential modulators separately. In 2013, Jansen et al. (39) performed the first meta-analysis in this field (both TMS and tDCS studies included). After comparing the effects on alcohol/nicotine users and people with a high craving for food, the results indicated that craving levels were decreased in substance dependence following non-invasive neurostimulation of the prefrontal cortex and further revealed a significant medium effect size of neurostimulation, but no difference between substances, stimulation technology, or side of stimulation. Recently, another meta-analysis from Song et al. (40) reported a medium effect size of TMS and tDCS treatment on craving and consumption, making a comparison between substance type/stimulation sessions/stimulated regions and found that multi-session stimulation had larger effects. Different from existing research, this meta-analysis evaluates tDCS based on separate stimulation parameters (e.g., the stimulation site, current intensity, number of sessions, duration of single-stimulation, and total-stimulation); the duration of stimulation, in particular, has seen little prior analysis. Substance type and study design may be important modulators that need to be investigated in depth. We aimed to conduct a meta-analysis focusing on the effects of tDCS in decreasing substance/food craving, and exploring the influence of potential modulators systematically. This should help find the optimal stimulation parameters in clinical settings for drug dependence and food addiction.

## Methods

An online search was conducted in the Web of Science, PubMed, and Google Scholar databases for articles published from January 2008 to January 2020. This search was implemented following the PICO-method (Patient/Population, Intervention, Comparison, Outcome) building a framework where “substance or food addiction” represented “P,” “tDCS stimulation” represented “I,” “active and sham stimulation” represented “C,” and “craving” represented “O”. The PRISMA (Preferred Reporting Items for Systematic Reviews and Meta-Analyses) guidelines were followed in this meta-analysis.

### Inclusion and Exclusion Criteria for the Selection of Studies

We searched for the combination of two sets of pre-defined terms in the title or abstracts. The following terms were set as the search terms: (transcranial direct current stimulation OR tDCS) AND (substance dependence OR substance abuse OR alcohol OR drug OR tobacco OR nicotine OR eating disorder OR food addiction).

All studies included in the meta-analysis met the following inclusion criteria: 1) tDCS was used as the stimulation tool; 2) substance craving/food craving changes were measured; 3) sham stimulation as a control condition; and 4) means, standard deviations, t, F, or p statistics and the number of participants in each intervention group were provided completely as basic data in order to calculate effect size.

Studies were excluded if: 1) they were meta-analyses, reviews, meeting abstracts, or case studies; 2) the subjects had other mental disorders other than substance addiction/food addiction; 3) tDCS was used combined with other intervention strategies (e.g., cognitive training or psychotherapy); or 4) the subjects were animals.

### Data Extraction

#### Stimulation Parameters

The most important stimulation parameters were stimulation site (left DLPFC, right DLPFC, or other area), current intensity (2 mA or 1 mA), number of stimulation sessions (single-session or multi-session), duration of single-stimulation (10–30 min), and total-stimulation (10–200 min).

#### Substance Type

Four types of substance dependence were analyzed: drugs (e.g., cocaine, marijuana, methamphetamine, heroin, and opium), tobacco, alcohol, and food.

#### Study Design

Studies included in this meta-analysis were divided into double-blind or single-blind experiments, and into parallel experiments (i.e., more than two groups of participants and each group receives different treatments) and crossover experiments (all participants receive the same treatments but in a random order).

#### Craving Measures

The main outcome measures included in this meta-analysis were state craving scores from different questionnaires. For each study, craving scores pre-tDCS and post-tDCS of all participants (both active and sham groups) were obtained. Effect sizes on craving levels were standardized for the effect of stimulation.

### Data Analysis

The Cochrane Collaboration's risk of bias tool was used to judge the risk of bias within individual studies (41). The publications identified through this search strategy were then examined by the three researchers (JQ, JC, and ZZ) individually to confirm eligibility. If there was any disagreement, it was resolved by discussion.

#### General Meta-Analysis

The Comprehensive Meta-Analysis 2.0 (CMA 2.0) computer program was used to process and analyze the data. Main analyses were implemented to examine the effects of tDCS on craving. To measure the difference in craving levels between active and sham stimulation, effect sizes (Hedges' *g*) were calculated. According to Borenstein et al. (42), Hedges' *g* is regarded as a conservative estimate, which could be applied to studies regardless of sample sizes. Basing on the value of *g,* the results may be defined as reflecting a small (g=0.2–0.5), medium (g=0.5–0.8), or large (g>0.8) effect.

#### Moderator Analyses

A random-effects model was used to analyze the difference between subgroups. Compared with the fixed effects model, the random effects model interval provides an unadventurous estimate of accuracy allowing for the existence of heterogeneity, which is more suitable for generalization (42). All comparisons used an alpha of 0.05.

The meta-regression was used to identify whether the duration of stimulation might influence the effect size estimates. Since every study was weighted by the precision of their respective effect estimates, the influence on the relationship depended on the size of study. This allows for residual heterogeneity among intervention effects rather than being modeled by explanatory variables (43).

#### Publication Bias

Publication bias analyzes whether the decision to publish or distribute a study was influenced by its findings (44). Publication bias was assessed using Egger's regression test (45). If publication bias was identified, a trim-and-fill procedure was applied to modify the effect size caused by publication bias (46).

## Results

### Studies Included in the Meta-Analysis

**Figure 1:**
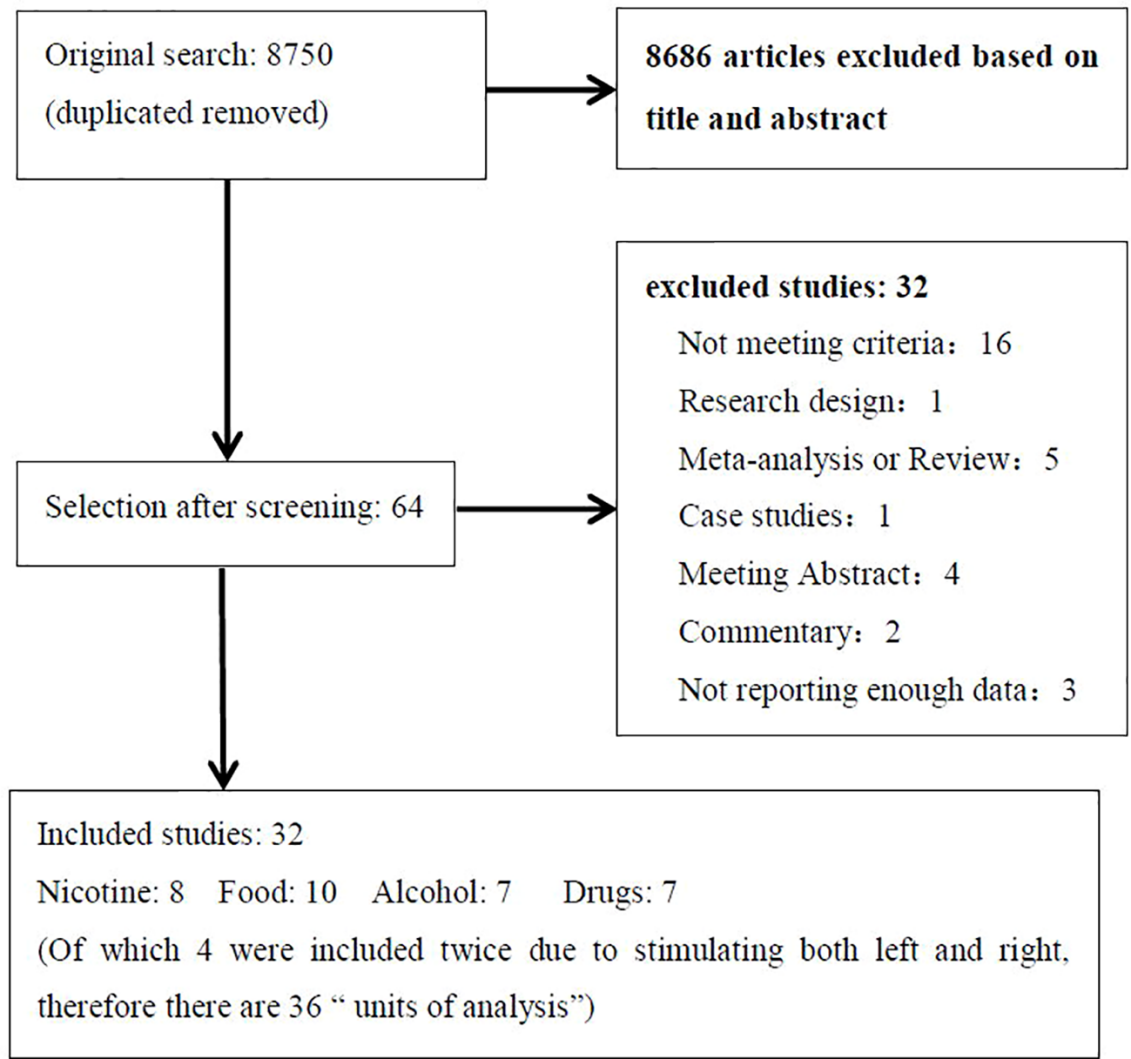
Flow diagram showing the search and selection procedure that was used for this meta-analysis. It showed the reasons for exclusion in “not meeting criteria” and final numbers of study included in the meta-analysis.

The literature search identified 32 eligible studies (see **Table 1**). **Figure 1** shows the flow diagram of the inclusion and exclusion process. Of the included studies, 8 for nicotine, 10 for alcohol, 7 for food, and 7 for other drug cravings. There were four studies in which subjects received two types of active anodal stimulation at different sites in independent sessions (33, 34, 57, 68). The four studies were divided into eight “units of analysis.” We adjusted for the interdependence of these data in the analyses by taking *study* as a unit of design instead of the *unit for analyses* because in this case it was difficult to distinguish differences in both design and stimulation locations (39). Therefore, there were 36 units of analysis included in total. The assessment of the risk of bias for all studies included is summarized in **Supplementary Table 1**.

**Table 1:** Studies included in the meta-analysis.

Author	Stimulation parameters				Study design			Participant's characteristics	
	Stimulation site	Current intensity	Sessions	Duration	Crossover or parallel	Double or single	Addiction type	Subjects	Craving measure
Fregni et al. (34)	A-R/L DLPFC	2 mA	1	20 min	CO	Double	Nicotine	24	VAS
Boggio et al. (47)	A-L DLPFC	2 mA	5	20 min	PR	Double	Nicotine	27	VAS
Fecteau et al.(48)	A-R DLPFC	2 mA	5	30 min	CO	Double	Nicotine	12	sQSU
Kroczek et al. (36)	A-L DLPFC	2 mA	1	15 min	PR	Double	Nicotine	25	VAS
Yang et al. (49)	A-L DLPFC DLPFC	1 mA	1	30 min	CO	Single	Nicotine	32	VAS
Xu et al. (38)	A-L DLPFC	2 mA	1	20 min	CO	Single	Nicotine	24	UTS
Mondino et al. (50)	A-R DLPFC	2 mA	10	20 min	PR	Double	Nicotine	29	LTS
Hajloo et al. (51)	A-L DLPFC	2 mA	10	20 min	PR	Double	Nicotine	40	DDQ
Boggio et al. (33)	A-R/L DLPFC	2 mA	1	20 min	CO	Double	Alcohol	13	AUQ
Silva et al. (52)	A-L DLPFC	2 mA	5	20 min	PR	Single	Alcohol	13	OCDS
Klauss et al. (53)	A-R DLPFC	2 mA	5	26 min	PR	Single	Alcohol	33	OCDS
den Uyl et al. (54)	A-L DLPFC	1 mA	1	10 min	PR	Single	Alcohol	41	AAAQ
Wietschorke et al. (55)	A-R DLPFC	1 mA	1	20 min	PR	Double	Alcohol	30	VAS
Klauss et al. (56)	A-R DLPFC	2 mA	10	20 min	PR	Double	Alcohol	49	OCDS
Nakamura-Palacios et al. (37)	A-L DLPFC	1 mA	1	10 min	CO	Single	Alcohol	49	OCDS
Fregni et al. (57)	A-R/L DLPFC	2 mA	1	20 min	CO	Double	Food	21	VAS
Goldman et al. (58)	A-R DLPFC	2 mA	1	20 min	CO	Single	Food	19	VAS
Kekic et al. (59)	A-R DLPFC	2 mA	1	20 min	CO	Double	Food	17	FCQ-S
Lapenta et al. (60)	A-R DLPFC	2 mA	1	20 min	CO	Double	Food	9	VAS
Jauch-Chara et al. (61)	A-R DLPFC	1 mA	8	20 min	CO	Single	Food	14	VAS
Georgii et al. (62)	A-R DLPFC	1 mA	1	20 min	CO	Double	Food	42	FCQ-S
Montenegro et al. (63)	A-L DLPFC	2 mA	1	20 min	CO	Single	Food	9	VAS
Ray et al. (64)	A-R DLPFC	2 mA	1	20 min	CO	Double	Food	18	VAS
Ray et al. (65)	A-R DLPFC	2 mA	1	20 min	PR	Single	Food	74	FCT
Chen et al. (66)	A-R IFG	1.5 mA	1	20 min	PR	Single	Food	57	FCQ-S
Batista et al. (67)	A-R DLPFC	2 mA	5	20 min	PR	Double	Cocaine	36	OCCS
Boggio et al. (68)	A-R/L DLPFC	2 mA	1	15 min	PR	Double	Marijuana	25	VAS
Shahbabaie et al. (69)	A-R DLPFC	2 mA	1	20 min	CO	Double	Meth	30	VAS
Wang et al. (70)	A-OL	1.5 mA	1	20 min	PR	Single	Heroin	20	VAS
Shahbabaie et al. (71)	A-R DLPFC	2 mA	1	26 min	CO	Double	Meth	15	VAS
Taremian et al. (72)	A-R DLPFC	2 mA	10	20 min	PR	Single	Opium	60	DDQ
Anaraki et al. (73)	A-R DLPFC	2 mA	5	20 min	PR	Single	Meth	30	DDQ

A-R, anodal-right; A-L, anodal-left; DLPFC, dorsolateral prefrontal cortex; OL, occipital lobe; IFG, inferior frontal gyrus; CO, crossover; PR, parallel; Meth, methamphetamine; VAS, Visual Analogue Scale; sQSU, standardized Questionnaire of Smoking Urges; OCCS, Obsessive-Compulsive Cocaine Scale; LTS, Likert-type scale; UTS, urge to smoke; DDQ, The Desire for Drug Questionnaire; AUQ, Alcohol Urge Questionnaire; OCDS, Obsessive Compulsive Drinking Scale; AAAQ, Alcohol Approach and Avoidance Questionnaire; FCQ-S, Food Craving Questionnaire State; FCT, Food Craving Task.

### General Effect Size (Hedges' *g*) on Craving

**Figure 2:**
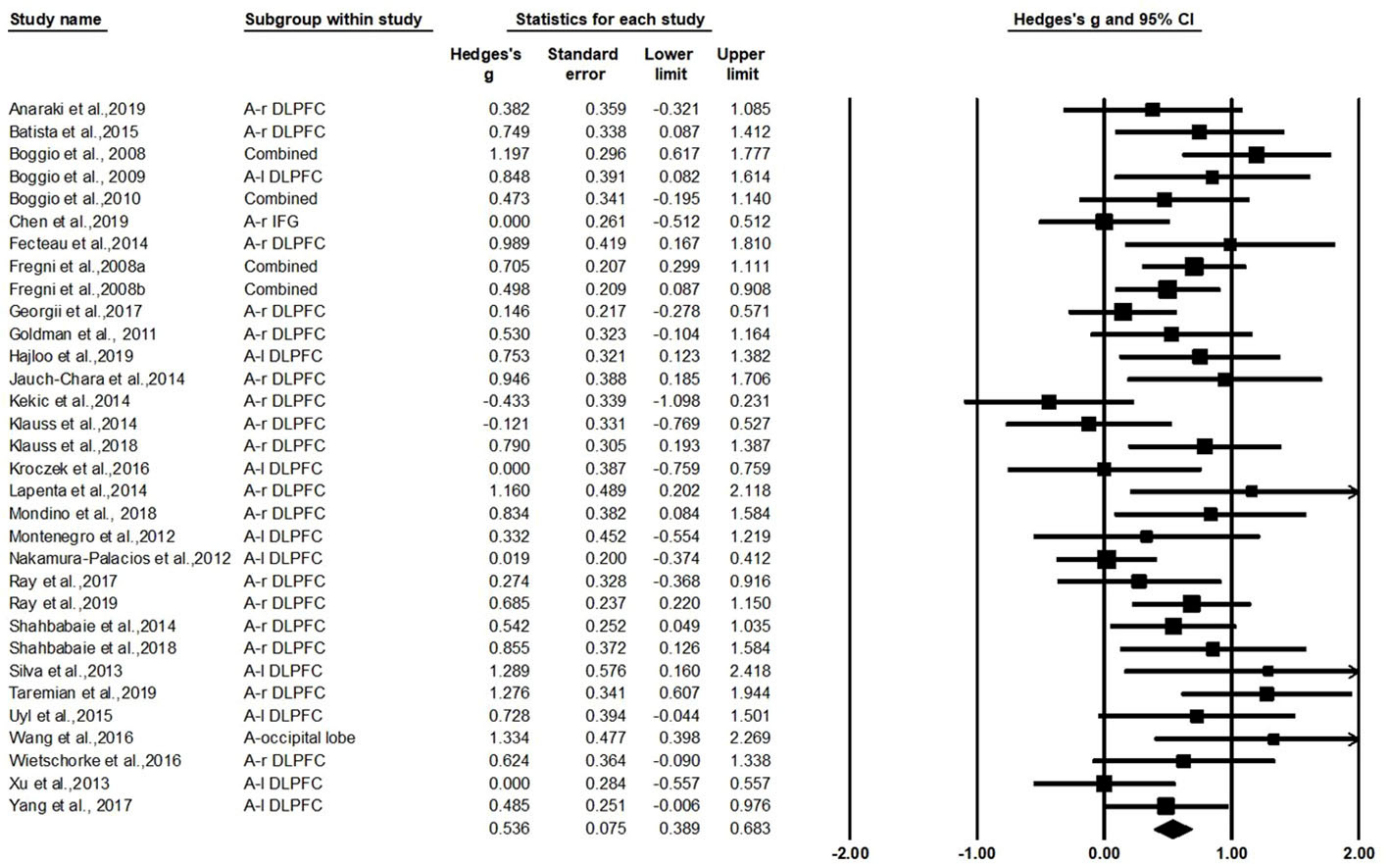
The overall effect of transcranial direct current stimulation (tDCS) on craving.

The test for heterogeneity was significant (Q=55.44, df=31, *p*=0.004, I^2^=44.08%), showing that there was heterogeneity between the study findings. In order to address the heterogeneity problem, we used a random effects analysis for the meta-analysis because of its conservative estimate and appropriate nature for generalization (42). As shown in **Figure 2**, this analysis revealed a standardized effect size (Hedges' *g*) of 0.536 [95% confidence interval (CI): 0.389–0.683], indicating a medium effect size favoring active stimulation over sham stimulation (z=7.153, p<0.001). The variation caused by the true difference in the effect accounts for 44.08% of the total variation with medium heterogeneity in this study, which implies that there may be important potential modulating variables and subgroup analysis was needed to test for the moderating effects (74, 75).

### Influence of Moderators

#### Stimulation Parameters (Stimulation Site, Current Intensity, Number of Sessions)

The left or right DLPFC was the anodal stimulation site in all studies except two (66, 70), which were excluded from this subgroup analysis, leaving 13 units for the left DLPFC and 21 units for the right DLPFC. Comparisons were made between the left and right DLPFC regardless of the cathodal site. The results showed that the difference between the left DLPFC and right DLPFC was not significant (Q=2.673, *p*=0.102) although Hedges' *g* for the left DLPFC was 0.402, while the Hedges' *g* for the right DLPFC was 0.636.

As far as current intensity was concerned, a comparison was made between 1 mA and 2 mA [six studies with 1 mA, 24 studies with 2 mA, two studies was not included because the stimulation current was 1.5 mA (66, 70)]. There was no significant difference in effect size between 1 and 2 mA (Q=1.635, *p*=0.201); the Hedges' *g* was 0.381 for 1 mA and 0.592 for 2 mA (**Table 2**).

**Table 2:** Results of subgroup analysis (random-effects model).

Moderators	*k*	Hedges' *g*	95% CI	Heterogeneity	
				*Q*_B_	*P*
**Stimulation site**				2.673	0.102
Left DLPFC	13	0.402***	[0.195, 0.609]		
Right DLPFC	21	0.613***	[0.446, 0.826]		
**Current intensity**				1.478	0.224
1 mA	6	0.381***	[0.102, 0.661]		
2 mA	24	0.583***	[0.417, 0.748]		
**Number of sessions**				4.261	0.039
Single-session	21	0.444***	[0.272, 0.615]		
Multi-session	11	0.751***	[0.515, 0.987]		
**Substances type**				4.121	0.249
Nicotine	8	0.555***	[0.309, 0.800]		
Alcohol	7	0.587***	[0.166, 1.008]		
Food	10	0.371***	[0.122, 0.620]		
Drugs	7	0.742***	[0.483, 1.000]		
**Double or single**				0.405	0.524
Double	18	0.582***	[0.404, 0.761]		
Single	14	0.483***	[0.236, 0.730]		
**Crossover or parallel**				0.913	0.339
Crossover	16	0.471***	[0.266, 0.677]		
Parallel	16	0.614***	[0.405, 0.823]		

***p < .01.

**Figure 3:**
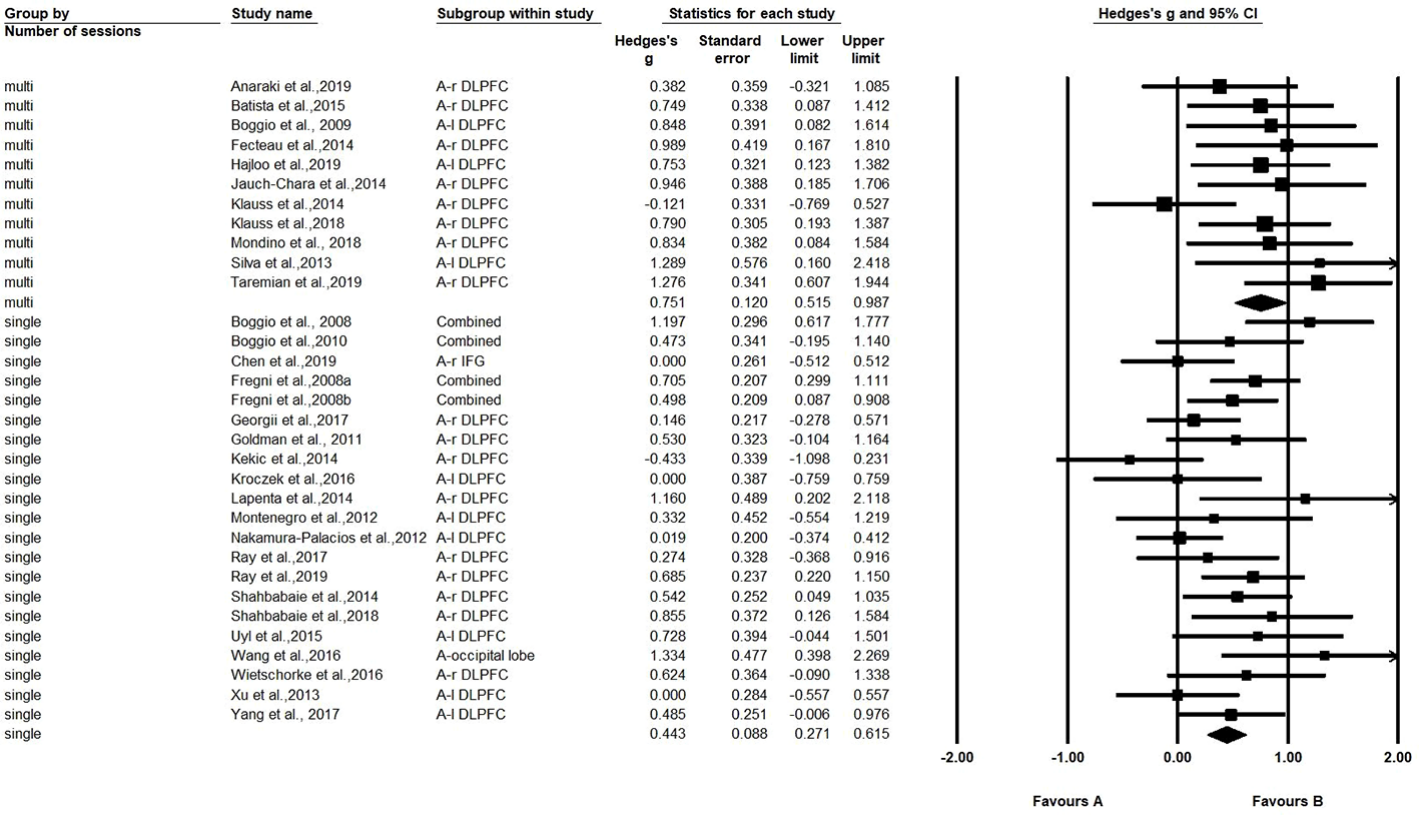
The difference between single session and multi-session.

A comparison was also made between the number of sessions (single-session *vs*. multi-session) (21 studies with one session, and 11 studies with multiple sessions). The Hedges' *g* for single-session stimulation was 0.443, while the Hedges' *g* for multi-session stimulation was 0.751 (see **Figure 3**). The results revealed a significant difference in effect size between the two subgroups (Q=4.261, *p*=0.039) (see **Table 2**). We found multiple session of tDCS intervention may have a better effect on craving.

#### Duration of Single Stimulation and All Stimulation

**Figure 4:**
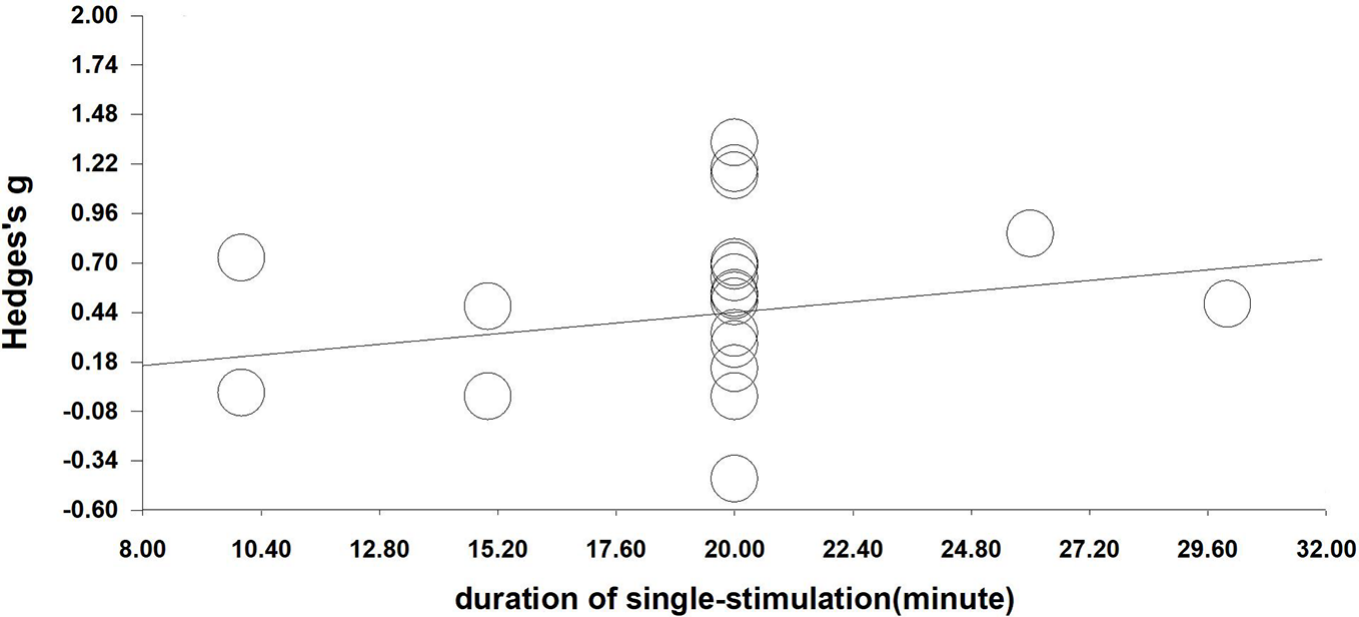
Regression of the duration of single-stimulation on the effect size of neuromodulation of craving. (Q=2.832, *β*=0.023, *p*=0.092).

**Figure 5:**
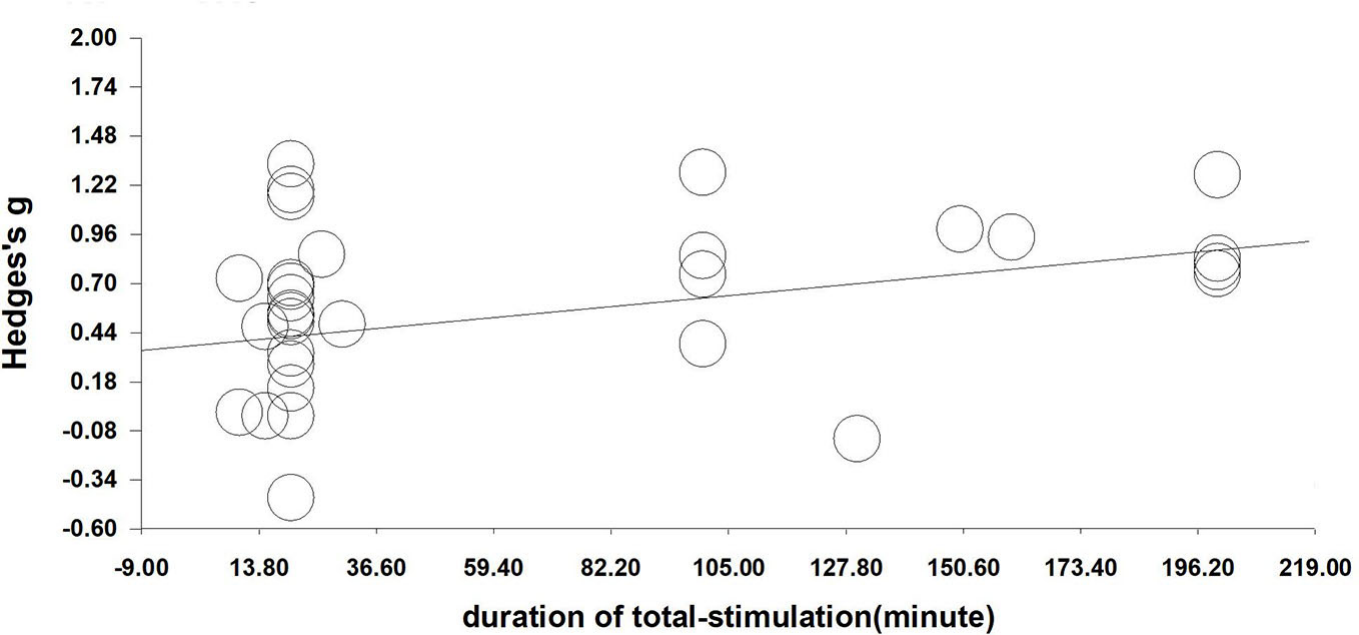
Regression of sessions on the effect size of neuromodulation of craving. (Q=8.505, *β*=0.003, *p*=0.004).

We performed a meta-regression analysis based on the duration of single stimulation and Hedges' *g*. Regarding the duration of single stimulation, we found a marginally significant result that the longer the stimulus lasts, the more the craving decreases (Q=2.832, *β*=0.023, *p*=0.092) (see **Figure 4**). Due to only the presence of only five values and the marginally significant result, we extended the analysis to include total stimulation time (duration of single stimulation × number of sessions) to obtain a more credible result. Regarding total stimulation duration, we found that longer stimulation was related to greater effect size (Q=8.505, *β*=0.003, *p*=0.004) (see **Figure 5**).

#### Substance Type

We compared the results for alcohol users, nicotine users, drug users, and food bingers. The results did not show a significant difference in effect size between substance types [Q (3)=4.121, *p*=0.249] (see **Table 2**).

When substance type was divided into two categories, substance and food, where alcohol, nicotine, and drugs were combined into substance, the Hedges' *g* for substance was 0.616, while the Hedges' *g* for food was 0.371. However, there was no significant difference between substance and food (Q=2.474, *p*=0.116).We also compared food with other substance types separately, and found that there was an obvious difference between drugs and food (Q=4.094, *p*=0.043), but no difference between alcohol and food nor nicotine and food.

#### Study Design (Crossover-Design or Parallel-Design; Double-Blind or Single-Blind)

A comparison was made between study designs. No significant difference was found between crossover and parallel designs (Q=0.913, *p*=0.339) or between double and single blind designs (Q=0.405, *p*=0.524) (see **Table 2**). However, we found that the number of sessions may influence the results because most (8/11) multiple stimulation studies were parallel designs. Therefore, we compared the effects of the crossover design and parallel design by using the number of sessions as a covariate. The result confirmed no significant difference in effect size between crossover and parallel designs (Q=1.77, *p*=0.183).

### Evaluation of Publication Bias

**Figure 6:**
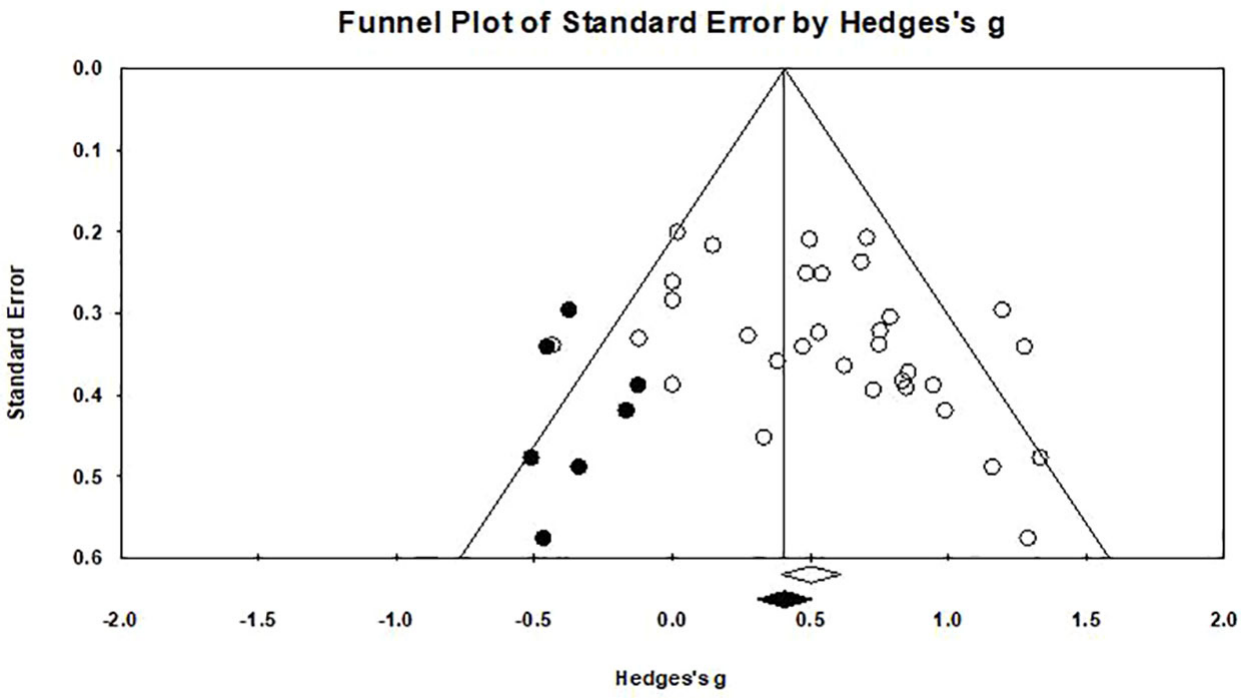
Funnel plot for a model without moderators (random-effects model), the solid circles in the funnel plot represents the seven studies that were trimmed to the left. The solid diamond on the abscissa indicate that the correction effect size is 0.416, and the hollow diamond on the right represents the original effect size is 0.536.

Egger's regression test was performed to empirically examine the presence of any publication bias. A publication bias was observed (*p*=0.031), as shown in **Figure 6**. A funnel plot was created (76) in which the measure of precision (standard error) of the effect size (Hedges' *g*) was shown with a trim-and-fill procedure applied (46). The results of the trim and fill method highlighted that there are seven “missing” effect sizes on the left side of the funnel plot. After adjustment, the analysis indicated an average effect size of 0.416 (95% CI: 0.262–0.570), which was comparable to the original result (Hedges' *g*=0.536), suggesting that the publication bias influenced our results lightly.

## Discussion

Based on 32 empirical studies, we performed a meta-analysis to review the effect of tDCS on substance craving and the influence of potential moderators. The results revealed a significant medium effect size (Hedges' *g*=0.536 or 0.416 after adjusting for publication bias) favoring real tDCS stimulation over sham stimulation in the reduction of craving. Furthermore, the number of sessions could significantly influence the effect of tDCS, favoring multi-session over single-session treatment. Other modulators appear to have no influence on craving reduction, such as stimulation site, current intensity, substances type, or study design.

### Effect of Transcranial Direct Current Stimulation on Reducing Craving and Its Possible Mechanism

Our findings confirmed that there was significant decrease in craving after tDCS stimulation of the DLPFC, which supports previous findings (39, 40, 77, 78).

One possible mechanism by which DLPFC stimulation decreases craving is an increase in “cognitive control.” As we know, the executive control network (ECN, including the DLPFC, orbitofrontal cortex, and anterior cingulate cortex) plays an important role in human executive control (79–81), including craving control (82, 83). tDCS can induce and regulate neural plasticity (84). Bilateral tDCS with anodal stimulation of the right DLPFC increases the intra-network functional connectivity (71). Cavaliere et al. (85) also showed that anodal tDCS over the DLPFC increases intra-network co-activation of the ECN. Thus, increased ECN activity and functional connectivity of the ECN could help individuals to decline or reduce craving—in other words, after changing the activity of the DLPFC subjects were able to better suppress their urges through its connections to the ECN.

Stimulation of the prefrontal cortex-stimulated dopaminergic pathways is another explanation. Addicted subjects were hardly roused by nondrug-related stimuli other than the substance they were addicted to, with decreased dopamine function disrupting frontal inhibition (86). Researchers have found that DLPFC stimulation alters the activation and functional connectivity of the cortical and subcortical reward systems in healthy individuals (87). Simultaneously, the membrane potential of pyramidal cells are regulated by anodal and cathodal tDCS, which alter the glutamate tone in the cortex, which is considered to correlate with GABA release, to restore the best excitation/inhibition balance to achieve the best steady-state plasticity in learning and cognition (88). Thus, tDCS stimulation of the DLPFC may improve the behavior of substance-dependent individuals by modulating the activities of brain regions such as the anterior cingulate cortex (ACC), the orbitofrontal cortex (OFC), and caudate nucleus.

However, it is not clear how tDCS affects substance and food craving. Further studies are needed to explore the psychological process and neural mechanisms underlying tDCS stimulation at the same time. A promising method can be seen in studies that use functional magnetic resonance imaging (fMRI) to explore the changes caused by tDCS stimulation (87, 89).

### Influence of Moderators

Among all the stimulation parameters, our subgroup analyses showed that multi-session tDCS had a significant greater effect on reducing craving than single-session tDCS, which is in agreement with Song et al.'s (40) and Kim et al.'s (77) findings. This suggests that the effects of tDCS on craving reduction can be cumulative. However, it is worth noting that the multiple stimulations included in the meta-analysis were all performed separately and not on the same day, because one study reported there was no benefit of twice-daily stimulation over once-daily stimulation in increasing cortical excitability (90).

Although not significant, we found a trending positive influence of the duration of single stimulations (*p*=0.092). The non-significant results may be because there were sufficient data in the analysis. After calculating the total stimulation duration, there was a trend that longer durations were related to larger Hedges' *g* (*p*=0.004). The result is consistent with the finding that the duration of stimulation may enhance its efficacy in given applications (91). However, more evidence of the effect of stimulation duration is needed in future.

According to the substance type subgroup analyses, the effects on drug users were the biggest (Hedges' *g*=0.742), followed by alcohol and nicotine (Hedges' *g*=0.587 and Hedges' *g*=0.555), and then food (Hedges' *g*=0.371), suggesting that the process of food addiction may differ from substance addiction. In some study, food addiction has a bearing on stress exposure with damage to the hippocampus (92) and patients with food addiction were easily affected by a sad mood (93). Stress, cognition, and emotion recognition may play a more important role in food addiction than other addictions meaning the treatment of food addiction may be more complex.

As for other factors, since the beneficial effects of tDCS vary with the pathology (94), we speculate that tDCS may be more effective for severely addictive substances than lightly addictive substances. That is to say that the severity of addiction may be a factor influencing the effect size of tDCS. However, the severity of addiction cannot be determined in the current study because there is no consistent standard for severity across various addictions and we have difficulty extracting effective data to distinguish the severity of addiction in the current literature.

Although we tried to explore various regulatory variables, there are still some possible influencing variables, which are not discussed in this paper, such as tDCS combined with other treatments (cognitive bias modification, emotional regulation). tDCS combined with cognitive bias modiﬁcation has shown limited effect on treatment outcomes (95–97). Moreover, emotional regulation can help the curative effects of tDCS (98–101); after emotion regulation training, using tDCS has better results (100). Thus, different substance addictions may affect the participants' emotional management, in turn altering the effects of tDCS. Due to the limited number of studies and the great heterogeneity among the literature, they were not included in the current meta-analysis. However, these works are of great significance and may be a new trend for tDCS research that deserves further research.

Regarding stimulation site, we found that there was no difference between right/anodal, left/cathodal and left/anodal, right/cathodal protocols for substance, and food dependence, supporting the conclusion of Jansen et al. (39) that putting the anode on the left or right would not affect the results of treating addictive disorders. However, although not significant, we could not ignore that the right DLPFC may be a better choice for most individuals because of the higher Hedges' *g* (0.613) than that of the left DLPFC (0.402). Boggio et al. (68) showed that only the right anode + left cathode montage was significantly associated with a reduction of craving for marijuana. It seems there may have the possibility that the effect of stimulation site will depend on the health status of the subject and the type of substance dependence. Other parameters, including current intensity (1 *vs*. 2 mA), study design (crossover or parallel, and double blind or single blind) had no significant difference.

### Limitations and Future Directions

In this meta-analysis, some limitations should be considered. First, the number of libraries and articles available was limited. Although we searched for accessible published articles, we still could not be sure that all relevant research was included. Second, the main effect of tDCS is only a medium effect size, suggesting more empirical studies are needed to explore the modulators of tDCS treatment of craving control, e.g., the substance type, addiction severity, and abstinence duration. Unfortunately, the majority of included studies did not report the abstinence duration or addiction severity; therefore, we could not examine its moderating effect in tDCS performed for craving control. Thus, an in-detail report of dependence-related data would be very important in the future. Third, the tools for measuring cravings need to be improved. In most studies, craving was measured using self-report questionnaires or visual analogue scales, which are subject to socially desirable answers. Some objective methods to measure drug craving (e.g., physiological measurement) would be very helpful in investigating the effect size of craving reduction.

## Conclusion

The current meta-analysis provided evidence that tDCS protocols improved the symptoms of substance and food dependence as indicated by reduced craving, but some modulators does influence the effect of tDCS treatment on addiction. In general, our findings suggested that 2 mA stimulation may be better than 1 mA; multi-session interventions may be better than single-session. We also suggested that tDCS may be not ideal for the treatment of food addiction, implicating that compared with substance addiction, food addiction may be different in nature and needs complex treatment such as tDCS combined with psychological interventions. In clinic, the optimal treatment plan for any specific type of addiction still needs be to be examined in depth in future.

## Data Availability Statement

All datasets generated for this study are included in the article/**Supplementary Material**.

## Author Contributions

Conceiving of the design framework: JC and ZZ. Writing the manuscript: JC and JQ. Revising the manuscript: JC, JQ, QH and ZZ.

## Funding

This work was supported by the Fundamental Research Funds for Central Universities (#SWU1809006).

## Conflict of Interest

The authors declare that the research was conducted in the absence of any commercial or financial relationships that could be construed as a potential conflict of interest.
