# Supplementary material for: A Meta-Analysis of Transcranial Direct Current Stimulation on Substance and Food Craving: What Effect Do Modulators Have?
Source: Front Psychiatry. 2020 Jun 26;11:598. doi: 10.3389/fpsyt.2020.00598 (PMC7332543; doi:10.3389/fpsyt.2020.00598)
Supplement: Supplementary file 1 [file Table_1.docx]

**Supplementary Table 1. Risk of Bias Assessment for Included Studies**

| **First Author** | **Random Allocation (Selection Bias)** | **Allocation Concealment (Selection Bias)** | **Blinding of Participants and Personnel (Performance Bias)** | **Blinding of Craving Outcome Assessments (Detection Bias)** | **Incomplete Outcome**  **Data (Attrition Bias)** | **Selective**  **Reporting** |
| --- | --- | --- | --- | --- | --- | --- |
| [1] Fregni et al.2008(a) | Low risk | Unclear risk | Low risk | Low risk | Low risk | Unclear risk |
| [2] Boggio et al.,2009 | Low risk | Unclear risk | Low risk | Low risk | Low risk | Unclear risk |
| [3] Fecteau et al.,2014 | Low risk | Low risk | Low risk | Low risk | Low risk | Unclear risk |
| [4] Kroczek et al.,2016 | Low risk | Unclear risk | Low risk | Low risk | Low risk | Unclear risk |
| [5] Yang et al.,2017 | Low risk | Unclear risk | Low risk | Low risk | Low risk | Unclear risk |
| [6] Xu et al.,2013 | Low risk | Unclear risk | Low risk | Low risk | Low risk | Unclear risk |
| [7] Mondino et al.,2018 | Low risk | Unclear risk | Low risk | Low risk | Low risk | Unclear risk |
| [8] Hajloo et al.,2019 | Low risk | Unclear risk | Low risk | Low risk | Low risk | Unclear risk |
| [9] Boggio et al.,2008 | Low risk | Unclear risk | Low risk | Low risk | Low risk | Unclear risk |
| [10] Silva et al.,2013 | Low risk | Unclear risk | Low risk | Low risk | Low risk | Unclear risk |
| [11] Klauss et al.,2014 | Low risk | Unclear risk | Low risk | Low risk | Low risk | Unclear risk |
| [12] Uyl et al.,2015 | Low risk | Unclear risk | Low risk | Low risk | Low risk | Unclear risk |
| [13] Wietschorke et al.,2016 | Low risk | Unclear risk | Low risk | Low risk | Low risk | Unclear risk |
| [14] Klauss et al.,2018 | Low risk | Low risk | Low risk | Low risk | Low risk | Unclear risk |
| [15] Nakamura-Palacios et al.,2012 | Low risk | Unclear risk | Low risk | Low risk | Low risk | Unclear risk |
| [16] Fregni et al.,2008(b) | Low risk | Unclear risk | Low risk | Low risk | Low risk | Unclear risk |
| [17] Goldman et al.,2011 | Low risk | Unclear risk | Low risk | Low risk | Low risk | Unclear risk |
| [18] Kekic et al.,2014 | Low risk | Low risk | Low risk | Low risk | Low risk | Unclear risk |
| [19] Lapenta et al.,2014 | Low risk | Low risk | Low risk | Low risk | Low risk | Unclear risk |
| [20] Jauch-Chara et al.,2014 | Low risk | Unclear risk | Low risk | Low risk | Low risk | Unclear risk |
| [21] Georgii et al.,2017 | Low risk | Unclear risk | Low risk | NA | Low risk | Unclear risk |
| [22] Montenegro et al.,2012 | Low risk | Unclear risk | Unclear risk | Low risk | Unclear risk | Unclear risk |
| [23] Ray et al.,2017 | Low risk | Unclear risk | Low risk | Low risk | Low risk | Unclear risk |
| [24] Ray et al.,2019 | Low risk | Unclear risk | Low risk | Low risk | Low risk | Unclear risk |
| [25] Chen et al.,2019 | Low risk | Unclear risk | Low risk | Low risk | Low risk | Unclear risk |
| [26] Batista et al.,2015 | Low risk | Low risk | Low risk | Low risk | Low risk | Unclear risk |
| [27] Boggio et al.,2010 | Low risk | Unclear risk | Low risk | Low risk | Low risk | Unclear risk |
| [28] Shahbabaie et al.,2014 | Low risk | Unclear risk | Low risk | Low risk | Low risk | Unclear risk |
| [29] Wang et al.2016 | Low risk | Unclear risk | Low risk | Low risk | Low risk | Unclear risk |
| [30] Shahbabaie et al.2018 | Low risk | Unclear risk | Low risk | Low risk | Low risk | Unclear risk |
| [31] Taremian et al.,2019 | Low risk | Unclear risk | Low risk | Low risk | Low risk | Unclear risk |
| [32] Anaraki et al.,2019 | Low risk | Unclear risk | Low risk | Low risk | Low risk | Unclear risk |

# References

[1] Fregni F, Liguori P, Fecteau S, Nitsche MA, Pascual-Leone A, Boggio PS.Cortical stimulation of the prefrontal cortex with transcranial direct current stimulation reduces cue-provoked smoking craving: a randomized, shamcontrolled study. J Clin Psychiatry (2008) 69(1):32–40. doi: 10.4088/JCP.v69n0105

[2] Boggio PS, Liguori P, Sultani N, Rezende L, Fecteau S, Fregni F. Cumulative priming effects of cortical stimulation on smoking cue-induced craving. Neuroscience letters. 2009 Sep 29;463(1):82-6. doi: 10.1016/j.neulet.2009.07.041

[3] Fecteau S, Agosta S, Hone-Blanchet A, Fregni F, Boggio P, Ciraulo D, Pascual-Leone A. Modulation of smoking and decision-making behaviors with transcranial direct current stimulation in tobacco smokers: a preliminary study. Drug and Alcohol Dependence. 2014 Jul 1;140:78-84.doi: 10.1016/j.drugalcdep.2014.03.036

[4] Kroczek AM, Häußinger FB, Rohe T, Schneider S, Plewnia C, Batra A, et al. Effects of transcranial direct current stimulation on craving, heart-rate variability and prefrontal hemodynamics during smoking cue exposure. Drug Alcohol Depend (2016) 168:123–7.doi:10.101 6/j.drugalcdep.2016.09.006

[5] Yang LZ, Shi B, Li H, Zhang W, Liu Y, Wang H, Zhou Y, Wang Y, Lv W, Ji X, Hudak J. Electrical stimulation reduces smokers’ craving by modulating the coupling between dorsal lateral prefrontal cortex and parahippocampal gyrus. Social Cognitive and Affective Neuroscience. 2017 Aug;12(8):1296-302.doi: 10.1093/scan/nsx055

[6] Jiansong Xu, Fregni F, Brody AL, Rahman AS. Transcranial direct current stimulation reduces negative affect but not cigarette craving in overnight abstinent smokers. Front Psychiatry (2013) 4:112. doi: 10.3389/fpsyt.2013.00112

[7] Mondino M, Luck D, Grot S, Januel D, Suaud-Chagny MF, Poulet E, Brunelin J. Effects of repeated transcranial direct current stimulation on smoking, craving and brain reactivity to smoking cues. Scientific reports. 2018 Jun 7;8(1):1-1.doi: 10.1038/s41598-018-27057-1

[8] Hajloo N, Pouresmali A, Goradel JA, Mowlaie M. The Effects of Transcranial Direct Current Stimulation of Dorsolateral Prefrontal Cortex on Reduction of Craving in Daily and Social Smokers. Iranian Journal of Psychiatry. 2019 Oct;14(4):291.doi: 10.18502/ijps.v14i4.1979

[9] Boggio PS, Sultani N, Fecteau S, Merabet L, Mecca T, Pascual-Leone A, et al. Prefrontal cortex modulation using transcranial DC stimulation reduces alcohol craving: a double-blind, sham-controlled study. Drug Alcohol Depend (2008) 92(1-3):55–60. doi: 10.1016/j.drugalcdep.2007.06.011

[10] da Silva MC, Conti CL, Klauss J, Alves LG, do Nascimento Cavalcante HM, Fregni F, Nitsche MA, Nakamura-Palacios EM. Behavioral effects of transcranial direct current stimulation (tDCS) induced dorsolateral prefrontal cortex plasticity in alcohol dependence. Journal of Physiology-Paris. 2013 Dec 1;107(6):493-502.doi: 10.1016/j.jphysparis.2013.07.003

[11] Klauss J, Penido Pinheiro LC, Silva Merlo BL, Correia Santos GD, Fregni F, Nitsche MA, Miyuki Nakamura-Palacios E. A randomized controlled trial of targeted prefrontal cortex modulation with tDCS in patients with alcohol dependence. International Journal of Neuropsychopharmacology. 2014 Nov 1;17(11):1793-803.doi: 10.1017/S1461145714000984

[12] den Uyl TE, Gladwin TE, Wiers RW. Transcranial direct current stimulation, implicit alcohol associations and craving. Biological psychology. 2015 Feb 1;105:37-42.doi: 10.1016/j.biopsycho.2014.12.004

[13] Wietschorke K, Lippold J, Jacob C, Polak T, Herrmann MJ. Transcranial direct current stimulation of the prefrontal cortex reduces cue-reactivity in alcohol-dependent patients. Journal of neural transmission. 2016 Oct 1;123(10):1173-8.doi: 10.1007/s00702-016-1541-6

[14] Klauss J, Anders QS, Felippe LV, Nitsche MA, Nakamura-Palacios EM. Multiple sessions of transcranial direct current stimulation (tDCS) reduced craving and relapses for alcohol use: A randomized placebo-controlled trial in alcohol use disorder. Frontiers in pharmacology. 2018 Jul 3;9:716. doi: 10.3389/fphar.2018.00716

[15] Nakamura-Palacios EM, de Almeida Benevides MC, da Penha Zago-Gomes M, de Oliveira RW, de Vasconcellos VF, de Castro LN, et al. Auditory eventrelated potentials (P3) and cognitive changes induced by frontal direct current stimulation in alcoholics according to Lesch alcoholism typology. Int J Neuropsychopharmacol (2012) 15(5) :601–16. doi : 10.1017/S1461145711001040

[16] Fregni F, Orsati F, Pedrosa W, Fecteau S, Tome FA, Nitsche MA, Mecca T, Macedo EC, Pascual-Leone A, Boggio PS. Transcranial direct current stimulation of the prefrontal cortex modulates the desire for specific foods. Appetite. 2008 Jul 1;51(1):34-41.

[17] Goldman RL, Borckardt JJ, Frohman HA, O’Neil PM, Madan A, Campbell LK, Budak A, George MS. Prefrontal cortex transcranial direct current stimulation (tDCS) temporarily reduces food cravings and increases the self-reported ability to resist food in adults with frequent food craving. Appetite. 2011 Jun 1;56(3):741-6.doi: 10.1016/j.appet.2011.02.013

[18] Kekic M, McClelland J, Campbell I, Nestler S, Rubia K, David AS, Schmidt U. The effects of prefrontal cortex transcranial direct current stimulation (tDCS) on food craving and temporal discounting in women with frequent food cravings. Appetite. 2014 Jul 1;78:55-62.doi: 10.1016/j.appet.2014.03.010

[19] Lapenta OM, Di Sierve K, de Macedo EC, Fregni F, Boggio PS. Transcranial direct current stimulation modulates ERP-indexed inhibitory control and reduces food consumption. Appetite. 2014 Dec 1;83:42-8.doi: 10.1016/j.appet.2014.08.005

[20] Jauch-Chara K, Kistenmacher A, Herzog N, Schwarz M, Schweiger U, Oltmanns KM. Repetitive electric brain stimulation reduces food intake in humans. The American journal of clinical nutrition. 2014 Oct 1;100(4):1003-9.doi: 10.3945/ajcn.113.075481

[21] Georgii C, Goldhofer P, Meule A, Richard A, Blechert J. Food craving, food choice and consumption: The role of impulsivity and sham-controlled tDCS stimulation of the right dlPFC. Physiology & behavior. 2017 Aug 1;177:20-6.doi: 10.1016/j.physbeh.2017.04.004

[22] Montenegro RA, Okano AH, Cunha FA, Gurgel JL, Fontes EB, Farinatti PT. Prefrontal cortex transcranial direct current stimulation associated with aerobic exercise change aspects of appetite sensation in overweight adults. Appetite. 2012 Feb 1;58(1):333-8.doi: 10.1016/j.appet.2011.11.008

[23] Ray MK, Sylvester MD, Osborn L, Helms J, Turan B, Burgess EE, Boggiano MM. The critical role of cognitive-based trait differences in transcranial direct current stimulation (tDCS) suppression of food craving and eating in frank obesity. Appetite. 2017 Sep 1;116:568-74.doi: 10.1016/j.appet.2017.05.046

[24] Ray MK, Sylvester MD, Helton A, Pittman BR, Wagstaff LE, McRae III TR, Turan B, Fontaine KR, Amthor FR, Boggiano MM. The effect of expectation on transcranial direct current stimulation (tDCS) to suppress food craving and eating in individuals with overweight and obesity. Appetite. 2019 May 1;136:1-7.doi: 10.1016/j.appet.2018.12.044

[25] Chen S, Jackson T, Dong D, Zhang X, Chen H. Exploring effects of singlesession anodal tDCS over the inferior frontal gyrus on responses to food cues and food cravings among highly disinhibited restrained eaters: A preliminary study. Neurosci Lett (2019) 706:211–6. doi: 10.1016/j.neulet.2019.05.035

[26] Batista EK, Klauss J, Fregni F, Nitsche MA, Nakamura-Palacios EM. A randomized placebo-controlled trial of targeted prefrontal cortex modulation with bilateral tDCS in patients with crack-cocaine dependence. International Journal of Neuropsychopharmacology. 2015 Nov 1;18(12):pyv066. doi: 10.1093/ijnp/pyv066

[27] Boggio PS, Zaghi S, Villani AB, Fecteau S, Pascual-Leone A, Fregni F. Modulation of risk-taking in marijuana users by transcranial direct current stimulation (tDCS) of the dorsolateral prefrontal cortex (DLPFC). Drug Alcohol Depend (2010) 112(3):220–5. doi: 10.1016/j.drugalcdep.2010.06.019

[28] Shahbabaie A, Golesorkhi M, Zamanian B, Ebrahimpoor M, Keshvari F, Nejati V, Fregni F, Ekhtiari H. State dependent effect of transcranial direct current stimulation (tDCS) on methamphetamine craving. International Journal of Neuropsychopharmacology. 2014 Oct 1;17(10):1591-8.doi: 10.1017/S1461145714000686

[29] Wang Y, Shen Y, Cao X, Shan C, Pan J, He H, et al. Transcranial direct current stimulation of the frontal-parietal-temporal area attenuates cue-induced craving for heroin. J Psychiatr Res (2016) 79:1–3. doi: 10.1016/j.jpsychires.2016.04.001

[30] Shahbabaie A, Ebrahimpoor M, Hariri A, . Nitsche MA, Hatami J, Fatemizadeh E, et al. Transcranial DC stimulation modifies functional connectivity of large-scale brain networks in abstinent methamphetamine users. Brain Behav (2018) 8(3):e00922. doi: 10.1002/brb3.922

[31] Taremian F, Nazari S, Moradveisi L, Moloodi R. Transcranial direct current stimulation on opium craving, depression, and anxiety: a preliminary study. The journal of ECT. 2019 Sep 1;35(3):201-6.doi: 10.1097/YCT.0000000000000568

[32] Rohani Anaraki M, Dolatshahi B, Nosratabadi M, Nouri Yalghouzaghaji M, Rezaei Mashhadi S. Repeated Transcranial Direct Current Stimulation (tDCS) on Methamphetamine Craving: A Randomized, Sham-controlled Study. Iranian Rehabilitation Journal. 2019 Dec 10;17(4):385-94.doi: 10.32598/irj.17.4.385
